# Prediction of Sentinel Node Status and Clinical Outcome in a Melanoma Centre

**DOI:** 10.1155/2013/904701

**Published:** 2013-12-25

**Authors:** Vera Teixeira, Ricardo Vieira, Inês Coutinho, Rita Cabral, David Serra, Maria José Julião, Maria Manuel Brites, Anabela Albuquerque, João Pedroso de Lima, Américo Figueiredo

**Affiliations:** ^1^Dermatology Department, Coimbra University Hospital, Praceta Mota Pinto, 3000-075 Coimbra, Portugal; ^2^Faculty of Medicine, University of Coimbra, 3000-075 Coimbra, Portugal; ^3^Pathology Department, Coimbra University Hospital, 3000-075 Coimbra, Portugal; ^4^Nuclear Medicine Department, Coimbra University Hospital, 3000-075 Coimbra, Portugal

## Abstract

*Background*. Sentinel lymph node biopsy (SLNB) is a standard procedure for patients with localized cutaneous melanoma. The National Comprehensive Cancer Network (NCCN) Melanoma Panel has reinforced the status of the sentinel lymph node (SLN) as an important prognostic factor for melanoma survival. We sought to identify predictive factors associated with a positive SLNB and overall survival in our population. *Methods*. We performed a retrospective chart review of 221 patients who have done a successful SLNB for melanoma between 2004 and 2010 at our department. Univariate and multivariate analyses were done. *Results*. The SLNB was positive in 48 patients (21.7%). Univariate analysis showed that male gender, increasing Breslow thickness, tumor type, and absence of tumor-infiltrating lymphocytes were significantly associated with a positive SLNB. Multivariate analysis confirmed that Breslow thickness and the absence of tumor-infiltrating lymphocytes are independently predictive of SLN metastasis. The 5-year survival rates were 53.1% for SLN positive patients and 88.2% for SLN negative patients. Breslow thickness and the SLN status independently predict overall survival. *Conclusions*. The risk factors for a positive SLNB are consistent with those found in the previous literature. In addition, the SLN status is a major determinant of survival, which highlights its importance in melanoma management.

## 1. Introduction

Sentinel lymph node biopsy (SLNB) is the standard practice for pathological staging in patients with localized melanoma in most melanoma centers worldwide [1, 2]. With a 20% likelihood of yielding positive results, it spares most patients to a complete lymph node dissection (CLND), a more invasive procedure [1–6].

Although several factors have been identified as predictors of a positive SLNB, only few have been proved to be independent predictors after adjusting for confounding variables. Breslow thickness is the most consistently reported and well-established predictor of sentinel lymph node (SLN) metastasis. Other reported predictive factors are age, gender, primary site, ulceration, tumor mitotic rate, Clark level, lymphovascular invasion, and absence of tumor-infiltrating lymphocytes [5–9]. SLN status is an important prognostic factor in melanoma patients [1]. According to this, management guidelines issued by the National Comprehensive Cancer Network (NCCN) emphasize the role of SLN biopsy as staging and prognostic procedure [10].

We investigated the association of several clinical and pathological variables with an increased likelihood of positive SLNB and factors that have an impact in melanoma-related death in our population.

## 2. Material and Methods

The study was approved by the Research Ethics Board of Coimbra University Hospital. We did a retrospective chart review of 221 cases of cutaneous melanoma which had a successful SLNB. The cases were all from our department and refer to the period from January 2004 to December 2010. The followup was extended to June 2012. The procedure was performed in the presence of melanoma >1.0 mm, or even thinner if adverse prognostic features were present, as recommended by NCCN guidelines. Most of the patients were staged up to T1b, but nine patients were in T1a, seven of them with exactly 1 mm Breslow thickness. Only patients without clinical or radiological evidence of nodal or distant metastases were selected to be submitted to SLNB.

## 3. SLN Biopsy Technique

Lymphoscintigraphy was performed the day before surgery by intradermal injection of technetium 99 m sulfur colloid around the primary lesion or biopsy site to identify lymphatic basins by gamma imaging. Single-photon emission computed tomography (SPECT) drainage was used in patient's complex lymphatic drainage. The site of the sentinel lymph node (hot spot) was marked on the skin. On the day of surgery, with the aid of a hand-held gamma probe, a 10–15 mm incision was made over the marked lymph node basin and after careful exploration of the tissue, the SLB was localized and excised. All nodes with radioactive counts exceeding 10% of the node with the highest radioactive count were removed and sent for histopathological analysis [11].

## 4. Data Collection

Our data contains patient characteristics like age, gender, and location of the primary lesion (categorized into four anatomic locations: head and neck, trunk, upper limb, and lower limb) and histological features of the primary melanoma such as Breslow thickness, tumor type, mitotic rate, ulceration, neurotropism, angioinvasion, and presence/absence of tumor-infiltrating lymphocytes.

A pathologist (Maria José Julião) reviewed the histological sections of all positive SLN in our data to exclude misinterpretations in the original pathology reports. The slides were stained with hematoxylin-eosin and immunohistochemistry examination involved S100 and HMB45. The following micromorphometric features were registered: SLN basin site, number of positive SLN, size of largest metastatic deposit in SLN (stratified into 2 groups: ≤1 mm and >1 mm), intranodal location of tumor deposits (subcapsular, parenchymal, both, or extensive), number of metastatic foci, and presence of extranodal invasion and perinodal lymphatic invasion. CLND positivity (when performed), melanoma recurrence (peritumoral skin), and survival outcomes were assessed.

## 5. Statistical Analysis

Statistical analysis was performed using Software Package for Statistical Science (SPSS for Windows, version 18.0, Chicago, IL, USA). Categorical data are presented as frequency (percentage) and continuous data are presented as mean ± standard deviation. For the comparison of categorical data, a Chi-square test was done.

We used univariate and multivariate logistic regressions to test the correlation of each variable with SLNB positivity. Odds ratios of the significant predictors are provided along with 95% confidence intervals (CI). Some histological variables were reported inconsistently and were not included in the data.

Overall survival (OS) was calculated from the SLNB to the date of death or last follow-up visit for all patients. Only deaths due to melanoma were considered “events.” Kaplan-Meier survival curves were compared with the logrank test; multivariate analysis was performed using a Cox regression model to estimate significant independent prognostic factors on survival. A test statistic with a *P* value < 0.05 was considered significant.

## 6. Results

### 6.1. Clinical and Pathological Features

Forty-eight (21.7%) out of 221 patients with localized primary melanoma were tested positive upon SLN biopsy (Table 1). CLND was performed in 44 patients (4 patients refused the procedure), showing additional metastases in 13 patients (29.5%) (Table 2). The mean age of the cohort was 59.3 years (range 18–88), and 61.5% (*N* = 136) were females. Forty-three percent of melanomas were located on the lower limbs and 21.3% of all melanomas were located in the feet. The average Breslow thickness was 3.08 mm (±2.88 mm), and ulceration was present in 46.7% of cases. Local recurrence was observed in 11% of patients, on average after 15.6 months of SLN biopsy (Table 3). Melanoma-related death occurred in 14.9% (*N* = 33). The median follow-up duration was 44 months (range 3–110).

### 6.2. Predictors of Positive SLN

Table 1 shows the descriptive statistics of the clinical and pathological differences between patients according to SLN status. The univariate logistic regression showed that patient's gender (male), tumor type, Breslow thickness, and the absence of tumor-infiltrating lymphocytes are associated with a higher likelihood of a positive SLNB (Table 1). Mean Breslow thickness in negative SLNB group was 2.60 mm compared to 4.74 mm in positive SLN group (*P* < 0.001). Only 13.8% of the patients with lymphocytic infiltrate in the primary lesion had SLN positive compared to 30.2% of the patients without lymphocytic infiltrate (*P* = 0.021). No significant correlation was found between SLN status, patient's age (even after stratification by age groups, data not shown), tumor location, and SLN basin.

The multivariate analysis showed that Breslow thickness and absence of tumor-infiltrating lymphocytes were independent predictors of positive SLNB (Table 4). Others variables were no longer statistically significant.

**Figure 1:**
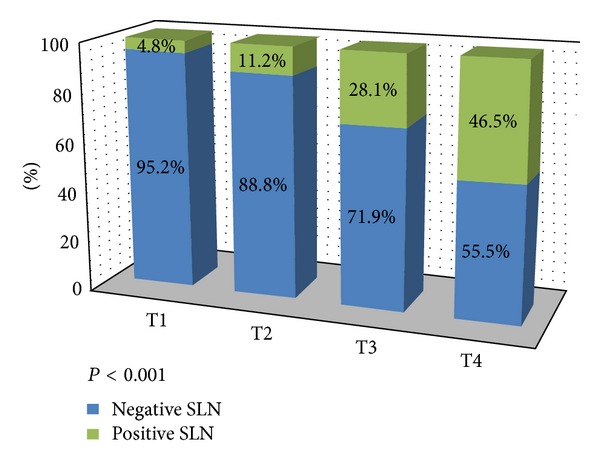
Association between Breslow category and SLN status.

The frequency of SLN metastasis is positively correlated with an increase in Breslow thickness: only one patient in T1 stage (4.8%) showed SLN involvement compared to almost half of the patients in T4 category (46.5%) (*P* < 0.001, Figure 1). For each additional mm in Breslow thickness, the likelihood of positive SLN increased by 12%.

### 6.3. Clinical Outcome

**Figure 2:**
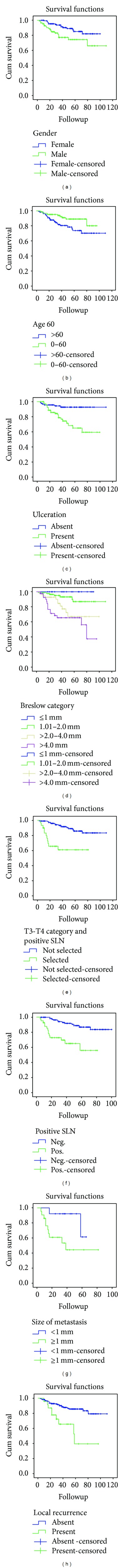
Association of melanoma-specific survival with clinical and pathological parameters. Survival estimates using Kaplan-Meier method; significance levels (*P* values) calculated using logrank tests. Not selected: all patients who are not positive SLN and T3 or T4 Breslow's category.

A Kaplan-Meier analysis identified a significant negative effect on overall survival of male gender (*P* < 0.05), age > 60 years (*P* < 0.05), ulceration (*P* < 0.001), increasing Breslow thickness (*P* < 0.001), positive SLNB (*P* < 0.001), maximum size of the largest tumor deposit >1 mm (*P* < 0.05), and local recurrence (*P* < 0.001) (Figure 2). We found no significant correlation between overall survival and extracapsular invasion, perinodal lymphatic involvement, intranodal location of tumor, number of metastatic foci, or CLND.

On multivariate Cox proportional hazard analyses, independent significant prognostic factors for melanoma-specific survival were Breslow thickness and SLN status, whilst the other variables lose their association (Table 5). The 5-year overall survival was significantly shorter in SLN positive patients than in SLN negative patients (53.1% versus 88.2%, *P* < 0.001), and about 35.4% of SLN positive patients (*N* = 17) had melanoma-related death compared with 9.2% SLN negative patients (*N* = 16) (*P* < 0.001, OR 5.38, 95% CI 2.46–11.78) (Table 2).

Of the 21 patients in T1 stage none died of melanoma (Table 6). Instead, the cases of melanoma-related death (MRD) increased with Breslow thickness (34.9% in T4 category, *P* < 0.001).

## 7. Discussion

Despite the small number of patients in our study, our major findings are consistent with previous large trials. In particular, the SLNB positivity rate and the percentage of additional lymph node metastasis reported in the previous literature are both about 20%, in line with our results [3, 5].

Some authors have questioned the role of SLN biopsy in melanoma management. Multicentre selective lymphadenectomy trial I (MSLT-I) showed that patients with positive SLNB who underwent immediate CLND had higher survival rates than those who only had lymph node dissection if clinical disease appeared (72% versus 52%) [1]. This result highlights the staging and prognostic value of SLN biopsy, with an attempted intervention when the nodal tumor burden in SLN positive patients is lower compared with clinically detected nodal metastases. Our results confirm the predictive significance of Breslow thickness and support the efficacy of SLN biopsy as a staging and prognostic procedure.

The previous literature shows conflicting results among predictors of positive SLNB. This reflects in part the heterogeneity in the measurement of the variables used in different studies, especially in the histological variables for which there is no standardized reporting [12].

Despite these findings, the practical indications for an SLNB have not substantially changed in the last years. Nowadays, an SLNB is formally recommended for patients over the stage IB in the AJCC melanoma staging system [10]. Stage IB includes cutaneous melanomas greater than 1 mm in thickness, or thinner melanomas that also have ulceration or at least 1 mitosis per millimeter squared [13]. An SLNB biopsy should also be discussed and considered for patients with stage IA (≤1 mm in Breslow thickness and no ulceration or mitoses) if adverse prognostic features are present [10]. Although there is no consensus about what defines “adverse prognostic features,” such features could include thickness over 0.75 mm, positive deep margins, lymphovascular invasion, or young age [10]. Although only 5% of positive SLNB results are found in T1 melanomas, a small group of patients will benefit from a therapeutic procedure such as CLND, or inclusion in control trials [14–16]. This explains our low threshold for an SLNB, and our results are consistent with other studies. As expected, we find that this subset of patients has a better prognosis, with no melanoma-related death among the 21 patients staged in T1.

The interaction between different factors is complex. Cadili and Dabbs (2010) found a higher rate of SLN metastasis in nodular melanoma, and they hypothesized an inherent biological characteristic of nodular melanomas as an explanation for this finding [12]. In addition, the presence of lymphocytic infiltrate was associated with a lower likelihood of a positive SLNB, which highlights its protector value against SLN metastasis [4, 17]. In contrast to other studies, we did not find any association of SLN positivity with age, even after stratification by age groups.

The SLN status was shown to be a highly significant prognostic factor of overall survival, with a 5-year survival of about 88% in patients with negative SLNB and 53% in those with positive SLNB. It is worth noting that factors predictive of SLN metastasis are similar to the prognostic factors for survival in melanoma patients [17]. The impact of SLN tumor features on survival is controversial. Some authors have demonstrated that the prognosis of SLN positive patients correlates with sentinel node tumor features, such as the maximum size of metastatic foci, intranodal location of tumor, extranodal spread, and perinodal lymphatic invasion [18–21]. Positivity of CLND was not significantly associated with a worse prognosis, perhaps due to the small sample in our study. To point out that the prognostic impact of local recurrence (peritumoral skin) was lost after adjustment for the others factors.

We share our clinical experience in 7 years of SLNB practice for cutaneous melanoma. This study has some limitations, as it is based on a relatively small sample and the variables were assessed retrospectively. Moreover, information about the mitotic rate, a recently T1b criterion [22], was not always present, and some incomplete histological reports did not allow to incorporate more variables for statistical treatment.

An interesting particularity in our melanoma patient's population is a remarkable high number of melanomas on the lower limbs, mainly on the feet (21.3% of all cases). The biological behavior of melanoma is influenced by numerous factors (genetic, environment) which can vary from one region to another. It is thus worth knowing more about our particular population, combining clinical and histological features, and identifying subgroups of patients to allow for an individual clinical decision supported by evidence-based guidelines.

## Figures and Tables

**Table 1:** Clinical and pathological features of patients who underwent sentinel lymph node biopsy (SLNB) between 2004 and 2010 by SLN status and univariable association with positive SLN.

	Total *N* (%)/mean (±SD)	SLN negative *N* (%)/mean (±SD)	SLN positive *N* (%)/mean (±SD)	*P* value	OR (95% CI)
Number of patients	221	173 (78.3)	48 (21.7)		
Age, yr	59.3 (±15.9)	58.8 (±16.4)	60.0 (±14.0)	NS	
Gender					
Female	136 (61.5)	116 (85.3)	20 (14.7)	<0.001	2.85 (1.48–5.49)
Male	85 (38.5)	57 (67.1)	28 (32.9)		
Tumor location					
Head and neck	23 (10.5)	20 (87)	3 (13)	NS
Upper limb	33 (15)	30 (90.9)	3 (9.1)	
Lower limb	95 (43)	70 (73.7)	25 (26.3)	
Trunk	69 (31.4)	52 (75.4)	17 (24.6)	
Histologic type					
Superficial spreading	38 (17.2)	35 (92.1)	3 (7.9)	0.001
Nodular	45 (20.4)	30 (66.7)	15 (33.3)	
Acral lentiginous	47 (21.3)	30 (63.8)	17 (36.2)	
Unknown/others (spitzoid, desmoplastic, nevoid, amelanotic)	91 (41.2)	78 (85.7)	13 (14.3)	
Breslow (mean, mm)	3.08 (±2.88)	2.60 (±3.88)	4.74 (±2.32)	<0.001	
Breslow category					
T1 (≤1 mm)	21 (10)	20 (95.2)	1 (4.8)	<0.001
T2 (1.01–2 mm)	89 (42.4)	79 (88.8)	10 (11.2)	
T3 (2.01–4 mm)	57 (27.1)	41 (71.9)	16 (28.1)	
T4 (≥4 mm)	43 (20.5)	23 (53.5)	20 (46.5)	
Ulceration					
Absent	97 (53.3)	79 (81.4)	18 (18.6)	NS
Present	85 (46.7)	60 (70.6)	25 (29.4)	
Tumor-infiltrating lymphocytes					
Present	80 (60.2)	69 (86.3)	11 (13.8)	0.021	2.71 (1.42–6.44)
Absent	53 (39.8)	37 (69.8)	16 (30.2)		
SLN site					
Axilla	92 (41.8)	72 (78.3)	20 (21.7)	NS
Inguinal	100 (45.5)	75 (75)	25 (25)	
Cervical	28 (12.7)	25 (89.3)	3 (10.7)	

NS: not statistically significant; CI: confidence interval.

**Table 2:** Micromorphometric features of SLN positive patients.

Micromorphometric features	N (%)
Size of metastasis	
≤1** **mm	15 (31.3)
>1 mm	24 (50.0)
Unknown	9 (18.7)
Intranodal location	
Subcapsular	16 (33.3)
Parenchymal	9 (18.7)
Both	15 (31.3)
Extensive	6 (12.5)
Unknown	2 (4.2)
Number of metastatic foci	
1	15 (35.7)
2–5	16 (38.1)
>5	11 (26.2)
Unknown	6 (12.5)
Extranodal invasion	
Presence	9 (19.1)
Lymphatic invasion	
Presence	10 (21.3)
CLND	
Positive	13 (29.5)

**Table 3:** Local recurrence and melanoma-related death.

	Total *N* (%)/mean (±SD)	Negative SLN *N* (%)/mean (±SD)	Positive SLN *N* (%)/mean (±SD)	OR (95% CI)	*P *value*
Local recurrence	24 (11)	8 (4.7)	16 (34.8)	10.93 (4.30–27.81)	<0.001^¥^
Time to local recurrence (median, months)	15.6 (±13.7)	17.8 (±19.0)	14.7 (±9.9)	—	NS^†^
Melanoma-related death	33 (14.9)	16 (9.2)	17 (35.4)	5.38 (2.46–11.78)	<0.001^¥^

NS: not statistically significant; CI: confidence interval; *P value of the Chi-square test (¥) or *t*-Student's test (†) as appropriate.

**Table 4:** Multivariate analysis of factors predicting a positive SLN.

Factor	Adjusted odds ratio (95% CI)	*P *value
Male gender	2.01 (0.77–5.22)	0.154
Breslow thickness (per mm)	1.12 (1.03–1.34)	0.020
Histologic type		
Superficial spreading	1.00*	
Nodular	3.35 (1.21–1.70)	0.193
Acral lentiginous	5.41 (1.69–3.44)	0.640
Unknown/others (spitzoid, desmoplastic, nevoid, amelanotic)	0.57 (0.57–0.27)	0.602
Tumor-infiltrating lymphocytes (absence)	2.77 (1.06–7.24)	0.038

CI: confidence interval; *this group served as the reference group.

**Table 5:** Cox multivariate analysis of the factors that were significant predictors of melanoma-related death.

Factor	Melanoma-related death	
	Hazard ratio (95% CI)	*P *value
Age (per yr of age)	1.011 (0.018–0.361)	NS
Gender (male versus female)	1.486 (0.450–0.774)	NS
Ulceration (present versus absent)	2.124 (0.541–1.937)	NS
Breslow thickness (per mm)	1.216 (1.118–1.323)	<0.001
SLN status (positive versus negative)	2.901 (1.254–6.713)	<0.001
Local recurrence	0.365 (0.591–2.914)	NS

CI: confidence interval; NS: not statistically significant.

**Table 6:** Melanoma-related death (MRD) and Breslow thickness.

Breslow category	Total (*N*)	MRD (*N*, %)
T1 (≤1 mm)	21	0
T2 (1.01–2.0 mm)	89	7 (7.9%)
T3 (2.01–4.0 mm)	57	11 (19.3%)
T4 (>4.0 mm)	43	15 (34.9%)

Total	210	33
